# Understanding Sleep Disturbances in Prostate Cancer—A Scientometric Analysis of Sleep Assessment, Aetiology, and Its Impact on Quality of Life

**DOI:** 10.3390/cancers15133485

**Published:** 2023-07-04

**Authors:** Stephen Mangar, Monica Abbadasari, Alessandro Carollo, Gianluca Esposito, Hashim Ahmed, Taimur Shah, Dagmara Dimitriou

**Affiliations:** 1 Department of Clinical Oncology, Imperial College Healthcare NHS Trust, Charing Cross Hospital, London W6 8RF, UK; s.mangar@imperial.ac.uk; 2 Sleep Education and Research Laboratory, UCL Institute of Education, London WC1H 0AA, UK; monica.abbadasari.18@ucl.ac.uk; 3 Department of Psychology and Cognitive Science, University of Trento, 38068 Rovereto, Italy; alessandro.carollo@unitn.it (A.C.); gianluca.esposito@unitn.it (G.E.); 4 Department of Surgery and Cancer, Imperial College London, London W6 8RF, UK; hashim.ahmed@imperial.ac.uk (H.A.); t.shah@imperial.ac.uk (T.S.); 5 Imperial Urology, Imperial College Healthcare NHS Trust, Charing Cross Hospital, London W2 1NY, UK

**Keywords:** prostate cancer, sleep, CiteSpace, scientometrics, bibliometrics, systematic review

## Abstract

**Simple Summary:**

Prostate cancer is the second most diagnosed cancer in men. It is driven by the male hormone testosterone, and measures used to block testosterone commonly known as hormonal therapy (Androgen Deprivation Therapy—ADT) forms an important treatment in localised and advanced disease. Patients who undergo ADT often experience fatigue and sleep disturbances which can affect quality of life resulting in poor compliance. This review of major publications relating to prostate cancer and sleep sought to gain an understanding of the methods used for sleep assessment and the magnitude of effect that ADT has on sleep. It highlights a lack of objective assessments, especially at baseline before ADT is commenced. It is recommended that future studies should employ a variety of methods for identifying and quantifying sleep disturbances. Implementing such methodology of assessment in future trials will help determine the impact that novel and established treatment will have on sleep quality.

**Abstract:**

Prostate cancer is the most commonly diagnosed cancer in the United Kingdom. While androgen-deprivation therapy is the most common treatment for prostate cancer, patients undergoing this treatment typically experience side effects in terms of sleep disturbances. However, the relation between prostate cancer and sleep and the way in which sleep interventions may benefit oncological patients is underinvestigated in the literature. The current study aims to review in a data-driven approach the existing literature on the field of prostate cancer and sleep to identify impactful documents and major thematic domains. To do so, a sample of 1547 documents was downloaded from Scopus, and a document co-citation analysis was conducted on CiteSpace software. In the literature, 12 main research domains were identified as well as 26 impactful documents. Research domains were examined regarding the link between prostate cancer and sleep, by taking into account variations in hormonal levels. A major gap in the literature was identified in the lack of use of objective assessment of sleep quality in patients with prostate cancer.

## 1. Introduction

Prostate cancer has now become the most commonly diagnosed cancer in the United Kingdom [1]. Whilst this is a result of earlier detection of localised disease, the prevalence of metastatic disease nevertheless remains a significant burden with approximately one in five patients presenting with advanced disease, together with a failure rate after primary radical treatment of up to 30% [2,3]. One of the most common treatments used in prostate cancer is androgen-deprivation therapy (ADT). It forms the mainstay of treating metastatic disease and is frequently used in combination with radiotherapy treatment in localised diseases to improve clinical outcomes. However, most patients experience side effects, with the most common complaints related to their sleep disturbances and reduced levels of daytime functioning. Whilst much is known about the long-term side effects, including the increased risk of metabolic syndrome, diabetes, cardiovascular complications, osteoporosis, and erectile dysfunction [4,5], it is the short-term vasomotor symptoms (VMS) together with sleep disturbances that can significantly impact quality of life and patient compliance of treatment [6].

However, scientific inquiry is complex here, as prostate cancer is usually diagnosed in older ages, which coincides with naturally occurring changes in sleep patterns with ageing, such as less efficient sleep, nap frequency and daytime sleepiness [7]. However, some recent epidemiological data suggest that excessive daytime sleepiness is not part of lifespan trajectory but is a sign of a medical condition. These data include chronic pain, arthritic conditions, diuretic medications resulting in frequent nocturnal urination, or mental health conditions such as depression and anxiety [8]. Poor quality and duration of sleep have been found to impact recovery from many different medical conditions [9]. Moreover, chronic diseases were reported to be more prevalent in adults who have a total sleep duration of under seven hours, with a direct correlation between decreased sleep and increased rates of chronic disease [10,11]. Hence, increasing our understanding of how to optimise sleep quality and quantity ought to be at the centre of investigatory research and clinical trials in common medical conditions such as prostate cancer. There is an aspiration in the field of sleep medicine which is undergoing a dynamic change in the use of new technology for assessing sleep as well in the area of sleep management and treatment in different populations.

The aim of this study was to identify the key publications on prostate cancer and sleep as well as the main thematic trends in a data-driven approach. In doing so, the study aims to also identify existing gaps in the literature. A document co-citation analysis (DCA) was conducted, and from the results of the scientometric analysis, publication trends and the connections between individual works were derived. DCA was chosen, as it provides an objective data-driven approach to identify thematic domains of research in large samples of documents.

## 2. Materials and Methods

### 2.1. Data Collection from Scopus

In line with the standard and established scientometric procedures [12,13,14,15,16,17,18,19,20,21,22,23], publications were downloaded from Scopus using the following search string TITLE-ABS-KEY (“prostate cancer” AND (“sleep” OR “insomnia”) AND (LIMIT-TO (LANGUAGE, “English”). The limitation of the search to English publications was used to allow for a more standardised and rigorous analysis of the work, as it will be built on the scientific literature on an international scale [24]. Data were collected on 25 January 2023. A total of 1547 documents published from 1981 to 2023 were found. A preliminary analysis of the citing documents was conducted by means of the bibliometrix package for R [25].

### 2.2. Data Eligibility

To conduct the scientometric analysis, data were imported into CiteSpace software (version 6.1.R6 64-bit Advanced) [26]. The software identified a total of 90,054 references in the documents collected from Scopus. Of the total number of references, 88,614 (98.40% of the total) were considered valid by the software. CiteSpace considers as valid only the references with all of the following seven key pieces of information: author, year of publication, title, source, volume, pages, and DOI [27]. A number of references being considered “invalid” were due to irregularities in the citation format. These negligible losses in references (1–5%) are a common occurrence due to incorrect citation formats when data are being imported into CiteSpace. The CiteSpace function Remove Alias was turned ON to remove identical or repeated entries [19].

### 2.3. Document Co-Citation Analysis (DCA)

A DCA was computed to determine the main research trends in the literature on prostate cancer and sleep. DCA relies on the frequency with which two or more documents are cited together in source publications [28]. The assumption of DCA is that common research domains are reflected by frequent co-citations among documents [29]. DCA generates a network that includes frequently co-cited documents together with the documents that cite them [23].

**Figure 1:**
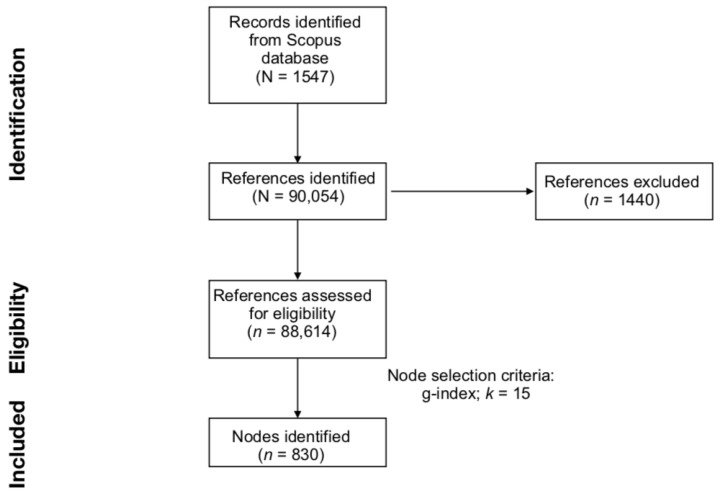
Preferred reporting items for systematic reviews (PRISMA) flowchart for literature search and references eligibility.

To identify a balanced network of documents, DCA parameters were optimised following the procedure of previous scientometric papers (e.g., [20,21,22,23]). To do so, several DCAs were computed. Each DCA was conducted with a different set of parameters (i.e., node selection criterion and scale factor). Three node selection criteria were used: *g*-index, TOP *N*, and TOP *N*%. Node selection criteria refer to a priori settings that determine the criteria for which documents are included in the final network. Particularly, the *g*-index is a measure of the citation scores of an author’s top publications [30,31]. The *g*-index value represents the largest number equal to the average number of citations of the most highly cited *g* publications [32]. Conversely, TOP *N* and TOP *N*% represent node selection criteria used to include, in the final network, the *N* and *N*% most cited documents within a time slice (i.e., one year), respectively [27]. For all DCAs, the time slice was maintained at the value of one year to retrieve the maximum amount of information [23]. Specific scale factor values are combined with the selected node selection criteria. Scale factors indicate the threshold to use for the respective node selection criteria [17]. In the current work, DCAs with the following node selection criteria and scale factor values were compared: *g*-index with scale factor *k* set at 10, 15, 20, 25, 35, and 50; TOP *N* with scale factor *N* set at 10, 25, and 50; and TOP *N*% with scale factor *N* set at 10. Values were selected and varied to optimise the structural properties of the network (i.e., modularity, silhouette, number of nodes, and number of major clusters), as recommended in the CiteSpace manual [27]. Ultimately, a DCA with a *g*-index with the scale factor *k* set to 15 was the parameter used to generate the final network of articles after these metrics were compared. The literature search and the generation of the DCA network are summarised in Figure 1.

### 2.4. DCA Network Evaluation Metrics

Two types of metrics were used in the description of the results from CiteSpace, namely structural and temporal metrics.

The group of structural metrics includes (i) modularity-Q, (ii) silhouette scores, and (iii) betweenness centrality. The modularity-Q represents the degree to which the network can be divided into single groups of nodes, which are referred to as modules or clusters. The modularity value ranges from 0 to 1, where higher values indicate a well-structured network [29]. Silhouette scores provide information on the consistency of clusters in terms of internal cohesion and separation from other clusters [33]. Values for silhouette score range from −1 to +1. Higher values are obtained by homogeneous clusters that are strongly separated from the rest of the network. Betweenness centrality represents the degree to which a node connects an arbitrary pair of nodes in the network [34]. Betweenness centrality ranges from 0 to 1, and higher values are obtained by ground-breaking documents.

Temporal metrics encompass (i) citation burstness and (ii) sigma. Citation burstness is computed by means of the Kleinberg’s algorithm [35]. The citation burstness represents an abrupt increase in the number of citations received by a document [36]. Finally, the sigma metric is an index for the novelty of a document and its influence on the overall network. Sigma is computed through the equation (centrality + 1)${}^{\mathrm{burstness}}$ [29].

## 3. Results

### 3.1. Bibliometric Analysis on the Citing Documents

The sample of documents collected from Scopus had an average of 53.08 citations per document. The countries appearing more frequently in the authors’ affiliation string were the United States of America (*n* = 605 documents; frequency = 0.4325; single-country publications (SCP) = 528; multiple-country publications (MCP) = 77), the United Kingdom (*n* = 89 documents; frequency = 0.0636; SCP = 57; MCP = 32), Canada (*n* = 88 documents; frequency = 0.0629; SCP = 69; MCP = 19), and Italy (*n* = 58 documents; frequency = 0.0415; SCP = 44; MCP = 14).

The most relevant sources for documents in the field of prostate cancer and sleep were Supportive Care in Cancer (*n* = 40 documents), the Journal of Clinical Urology (*n* = 35 documents), and the Journal of Urology (*n* = 30 documents).

In the sample, a total of 8155 unique authors were identified, with an average of 6.48 authors per document and 0.19 documents per author. The most productive authors in the network were C. Miaskowski, J. Savard, and S.M. Paul with 18, 17, and 16 documents published, respectively.

The citing documents with the highest number of citations were Haslam [37] (total citations = 3507; total citations per year = 184.6) and Parker et al. [38] (total citations = 2263; total citations per year = 205.7).

**Figure 2:**
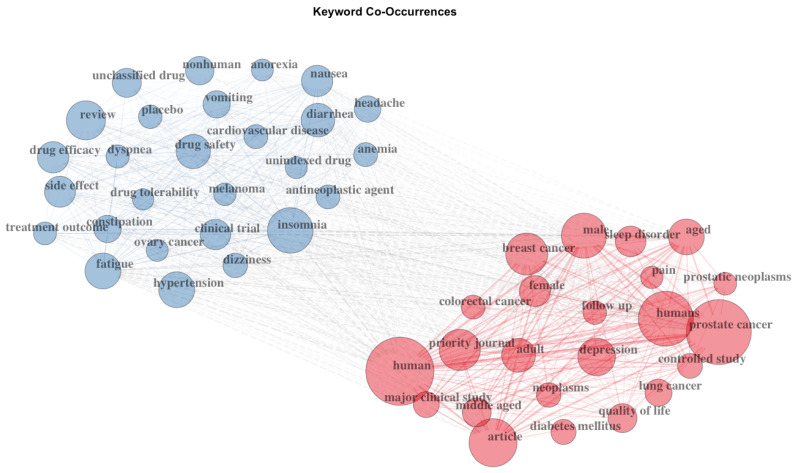
Top 50 keyword co-occurrences.

A total of 2791 unique keywords were identified in the documents. The most popular were prostate cancer (number of documents = 225), cancer (number of documents = 164), quality of life (number of documents = 107), testosterone (number of documents = 71), hypogonadism (number of documents = 53), fatigue (number of documents = 50), breast cancer (number of documents = 45), depression (number of documents = 43), sleep (number of documents = 40), and melatonin (number of documents = 36; see Figure 2 for the patterns of co-occurrence between the 50 most frequent keywords).

### 3.2. Structural Properties of the Document Co-Citation Analysis Network

**Figure 3:**
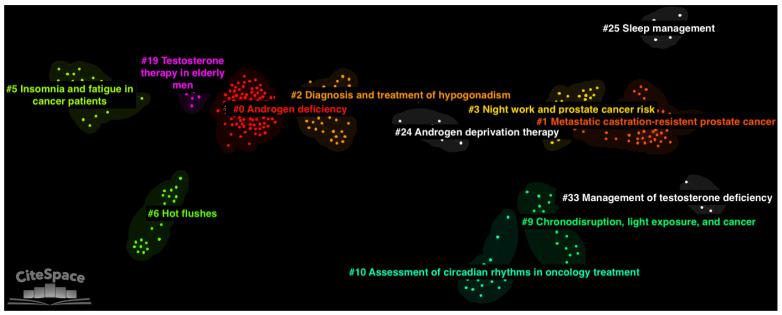
Document co-citation analysis network of the literature on prostate cancer and sleep generated with CiteSpace software version 6.1.R6 (64-bit Advanced) [26]. In the network, single nodes represent individual documents. Nodes are grouped into 12 main thematic clusters, which are depicted by colour in the image.

The final optimised network from the DCA consisted of 830 nodes and 2688 links, which indicates an average of 3.24 connections with other references for each node (see Figure 3). The network had a modularity Q index of 0.892 and a mean silhouette score of 0.971. These metrics indicate high divisibility into homogeneous clusters of the network.

### 3.3. Citation Burstness

In the network, a total of 26 documents were found to exhibit a citation burstness (see Table 1 for the top ten documents). The article with the highest burst strength was authored by Scher et al. [39] (strength = 9.58). The burst began in 2013 and lasted 6 years until 2019. This article investigated whether there is an increased survival benefit with enzalutamide in prostate cancer after chemotherapy. Scher et al. [39] found that median overall survival was 4.8 months higher for those who took enzalutamide compared to the placebo; however, rates of fatigue and hot flashes were higher. The next articles with the highest burst strength were Bhasin et al. [40] (strength = 8.45), which looked at testosterone therapy in men with androgen deficiency syndromes, and Beer et al. [41] (strength = 7.75), who wrote an article on enzalutamide in metastatic prostate cancer before chemotherapy. Beer et al. [41]’s article also had the longest burst duration, lasting 8 years from 2015 to 2023. They found that radiographic progression-free survival at 12 months was 51 percent higher with those who took enzalutamide compared to those who took a placebo; however, the adverse effects associated with enzalutamide use were hypertension and fatigue. Enzalutamide both increased radiographic progression-free survival and delayed the initiation of chemotherapy.

### 3.4. Thematic Clusters

A total of 12 major thematic clusters were identified in the final network of documents (see Table 2). A label was generated for each cluster using CiteSpace’s log likelihood ratio (LLR) algorithm. LLR was chosen because it is the most accurate among the automated labelling methods in CiteSpace. However, we conducted a visual inspection of clusters’ documents and amended the clusters’ labels when LLR lacked accuracy (as in [12]). The largest three clusters in the network were cluster #0 (size = 100; silhouette = 0.969; mean year of publication = 2002), #1 (size = 58; silhouette = 0.931; mean year of publication = 2013) and #2 (size = 50; silhouette = 0.990; mean year of publication = 2008). The most homogenous clusters due to their highest silhouette scores were clusters #33 (silhouette = 1.000; size = 6; mean year of publication = 2017), #25 (silhouette = 0.998; size = 7; mean year of publication: 2015) and #6 (silhouette = 0.996; size = 24; mean year of publication = 2000). The clusters with the oldest mean year of publication were clusters #19 (size = 11; silhouette = 0.99; average year of publication = 1994), #6 (average year of publication = 2000), and #5 (size = 26; silhouette = 0.985; average year of publication = 2000). The clusters with the most recent mean year of publication were clusters #33 (average year of publication = 2017), #3 (average year of publication = 2015), and #25 (average year of publication = 2015).

## 4. Discussion

This section will go through each cluster in detail, following the clusters’ clinical relevance. We will include the citing articles and the cited references within each cluster. In each cluster, the primary citing articles, along with their coverage and global citing score (GCS), will be highlighted in the analysis. Coverage is the number of articles in the cluster that the citing article cited, and GCS is the total number of citations a paper has acquired as recorded on Scopus. Only clusters that focus on sleep will be discussed in detail.

### 4.1. Cluster #5: Insomnia and Fatigue in Cancer Patients

The major citing articles in this cluster were written by Lee et al. [49], with a coverage of eight articles and a GCS of 136, Hervouet et al. [50] with a coverage of seven articles and a GCS of 61, and Simeit et al. [51] with a coverage of five articles and a GCS of 95. This cluster focuses on the relationship between cancer and sleep disturbances as well as possible therapies for this problem. According to Lee et al. [49], future studies on sleep and cancer must be specific to the type of cancer, gender, past history of sleep problems, treatment type, and sleep problem type. According to Lee et al’s investigation of the relationship between radiation therapy and sleep disturbances in prostate cancer patients, the most frequent sleep disturbances were frequent voiding and hot flashes. This kept patients awake and often was the worst during week 4 of the nine-week radiotherapy course. Hervouet et al. [50] studied 861 men with prostate cancer and their psychological struggles with various treatment techniques. Sleeping issues were discovered to be the second most often reported issue in the sample (31.9%). There was no discernible difference in levels of insomnia across the various treatment modalities (radiotherapy, brachytherapy, and radical prostatectomy), but levels of fatigue were higher in individuals who had additionally undergone hormone therapy.

There were three cited references that looked at cancer-related fatigue, sleep, and quality of life [52,53,54,55]. In particular, Savard and Morin [52] reviewed the evidence on the diagnosis, epidemiology, aetiology, and treatment of insomnia in the context of cancer and concluded that a multimodal approach to therapy, including both pharmacological and psychological therapies, would be appropriate because insomnia in cancer patients is caused by a variety of factors.

Possible treatments for sleep disturbances in relation to cancer were also addressed in this cluster. Many citing and cited articles in this cluster analysed different pharmacological, psychological, and exercise therapies for fatigue and sleep in cancer patients [51,56,57,58,59,60,61]. A study by Simeit et al. [51] investigated the advantages of sleep management training for cancer patients who also struggle with insomnia. They suggested that adopting a multimodal sleep intervention helped improve patients’ well-being and a variety of sleep parameters. In a review, Watson and Mock [60] suggested that exercise might play a role as an adjunct therapy for cancer-related fatigue. This demonstrated the importance of multimodal sleep therapies in cancer patients with sleep disorders.

### 4.2. Cluster #1: Metastatic Castration-Resistant Prostate Cancer

The main citing articles in this cluster were reports on randomised phase-three clinical trials investigating chemotherapy efficacy in metastatic castrate-resistant prostate cancer written by Oudard et al. [62] and Beer et al. [63]. They had a GCS of 191 and 56 and a coverage of 15 and 14, respectively. In the trial by Oudard et al. [62], 1167 patients were randomised to receive Docetaxel chemotherapy versus two different dose schedules (20 and 25 mg/m${}^{2}$) of Cabazitaxel. Overall (28–32%), grade three or greater levels of fatigue (1.6–2.8%) was recorded using the FACT-P assessment, with the dose level of 20 mg/m${}^{2}$ being roughly equivalent to Docetaxel, which was considered the gold standard. In the trial by Beer et al. [63], Custersin, a molecule that augments the biological response to Cabzitaxel, was added and tested against Cabazitaxel alone. This trial randomised over 630 patients but found no survival benefit from the addition of Custersin. In much the same way as Oudard et al. [62], fatigue was assessed using a health-related questionnaire, with grade three or greater occurring in approximately 6–7% of cases irrespective of the randomisation. No objective sleep assessments or sleep-specific questionnaires were utilised.

Another citing article in this cluster was written by Sparasci et al. [64]. They reviewed sleep disturbances and prostate cancer, paying particular attention to prostate cancer treatments and any potential sleep-related effects. The assessment covered 16 trials that took place between 1990 and 2021. Of the sixteen studies, none were randomised, and nine were prospective with study participants ranging from 24–263. Only five studies utilised actigraphy with numbers ranging from 24–99. Only three out of the five actigraphy studies looked specifically at ADT, and only one had a baseline assessment with a second time point at 12 months. In 14 of the 16 trials, patients receiving therapy for prostate cancer either developed a sleep disorder or their sleep patterns changed. The authors noted that in all large phase-three trials investigating novel hormonal agents plus ADT, compared to placebo or standard of care (SOC), sleep disorders have not been reported or assessed as independent adverse events. An exception to this is for trials involving the CYP17 inhibitor Abiraterone, where sleep problems and fatigue were noted in 1 and 2% of patients, respectively.

Sparasci et al. [64] drew attention to limitations such as the small number of existing research and, in particular, the heterogeneity of methodology. Further objective studies using methods such as actigraphy are therefore required to clarify the prevalence and significance of sleep problems brought on by prostate cancer treatments.

### 4.3. Cluster #10: Assessment of Circadian Rhythms in Oncology Treatments

The major citing articles in this cluster were written by Huang et al. [65], with a GCS of 5 and a coverage of 10, Madsen et al. [66] with a GCS of 26 and coverage of 8, and Savard et al. [67] with a GCS of 115 and coverage of 5. Savard et al. [67] conducted a population-based epidemiological study on the side effects of cancer treatments in breast and prostate cancers. It was found that the levels of insomnia, quantified by the insomnia severity index (ISI) [68], were higher in patients with prostate cancer who underwent hormone therapy. When hormone exposure was at its peak, the ISI scores were at their highest. Huang et al. [65] and Madsen et al. [66] reviewed several studies which used actigraphy to measure circadian rhythms during oncological treatments. They discovered that patients receiving a range of cancer therapies frequently experienced circadian rhythm disruption; yet light and behavioural therapy had some alleviating effects in patients with breast cancer. They highlighted the significance of minimising circadian disruption to improve the quality of life in patients. Madsen et al. [66] acknowledged that the majority of actigraphy had been completed in patients with breast cancer and that more research was needed for various other types of cancer such as prostate cancer. Two of the cited articles in the cluster investigated treatments for cancer-related sleep disturbances. A randomised controlled trial was carried out by Berger et al. [69] to see whether behavioural therapy can lessen cancer-related sleep disruption. Actigraphy and the Pittsburgh Sleep Quality Index (PQSI) were also employed. Behavioural therapy enhanced patients’ subjective ratings of their sleep quality, although actigraphy remained unchanged. To ascertain the effectiveness of cognitive behavioural therapy (CBT) for insomnia in breast cancer patients, Epstein [70] undertook a randomised controlled trial. Based on daily sleep diaries, CBT increased subjective sleep quality. These findings demonstrate that there is little research on non-pharmacological interventions for cancer-related sleep disturbances that use objective sleep metrics. Madsen et al. [66] also found this to be the case and recommended more research using actigraphy to evaluate the efficacy of treatments such as CBT for cancer-related sleep disorders.

### 4.4. Cluster #25: Sleep Management

There were two main citing articles in this cluster. The first was written by Delpachitra et al. [71], with a GCS of 4 and a coverage of 5. The second was written by Feng et al. [72], with a GCS of 6 and coverage of 3. Delpachitra et al. [71] used a brief online survey to explore ways in which patients with prostate cancer prefer to manage their sleep. To determine the severity of insomnia, the Insomnia Severity Index (ISI) was utilised. They discovered that past ADT usage and the severity of insomnia were significant predictors of the utilisation of sleep treatments. They concluded that patients with higher ISI scores were more likely to have utilised or be willing to employ sleep treatments. CBT and hypnotherapy were the two most popular therapies. To comprehend how ADT affects cancer-related fatigue and sleep, Feng et al. [72] looked at metabolomic alterations. Prior to radiotherapy, they took plasma samples from 160 participants—160 with and 160 without ADT as an adjunct—and used a Patient-Reported Outcome Measurement Information System (PRQMIS) to assess fatigue and sleep disturbances. It was discovered that patients who underwent ADT reported more severe levels of fatigue and that this fatigue is specifically related to sleep disruptions brought on by alterations in steroid hormone biosynthesis.

Further cited articles in this cluster also looked at cancer- and ADT-related sleep disturbances and possible interventions [73,74,75,76]. Gonzalez et al. [73] compared subjective (ISI) and objective (actigraphy) sleep data in patients with prostate cancer. Patients were assessed before starting ADT and at specific points after starting ADT. ADT patients reported higher levels of clinically significant sleep disturbances and hot flash interference. This was further demonstrated in individuals who underwent actigraphy, where those with ADT had higher levels of objective sleep disruptions and more frequent nocturia episodes. This study shows that hot flashes and nocturia can contribute to sleep difficulties in people receiving ADT.

A systematic study of the evaluation and treatment of cancer-related sleep was undertaken by Howell et al. [74]. The treatment techniques that were evaluated included exercise interventions, pharmaceutical interventions, sleep hygiene, and behavioural therapy (CBT). Evidence from randomised controlled trials (RCTs) indicated that CBT improved sleep disturbances in cancer patients. Although CBT was advised as the first line of treatment for individuals with insomnia in cancer, Howell et al. [74] discovered that they were unable to draw any conclusions about its superiority to alternative strategies. This was due to the heterogeneous nature of the studies on various interventions, such as pharmaceutical or exercise therapies. Howell et al. [74] recommended that further high-quality research be conducted into other interventions with a clear identification of insomnia and research that builds on existing dose and intervention components.

### 4.5. Cluster #3: Night Work and Prostate Cancer Risk

The two main citing articles in this cluster were written by Deng et al. [77] and Deng et al. [78], both of which were essentially a review of the literature. They have a GCS of 16 and 38, and a coverage of 16 and 13, respectively. The next two were written by Wendeu-Foyet et al. [79] and Turner et al. [80], which had a GCS of 27 and 0 and a coverage of 13 and 6, respectively. In their review, Deng et al. [77] found that shift work carries a moderate risk of developing prostate cancer. Nevertheless, at the time of their evaluation, no study had examined the impact of shift work sleep disorders on prostate cancer risk. To learn more about the relationship between night work and prostate cancer, Wendeu-Foyet et al. [79] undertook a case-control study among the French population. This study indicated that, while regular night work for a minimum of 20 years, with regularly working more than 6 consecutive nights or for more than 10 h, was shown to relate to aggressive prostate cancer, night work was not typically linked to the disease. A meta-analysis on sleep and cancer was carried out by Erren et al. [81] involving 1.5 million people in 13 different countries. The combined adjusted relative risk of sleep and circadian disruption in developing prostate cancer was 1.08. To be able to respond to the question “how are sleep and cancer linked”, Erren et al. [81] made suggestions for future research in their study. The requirement for a “standard” for examining associations between sleep and cancer was one significant recommendation. These standards should be supported by data from studies on chronobiological sleep and cancer. Doing so will reduce the sizeable heterogeneity between studies and boost the strength of combined studies. Further citing articles included a review by Porcacchia et al. [82] on sleep disorders and prostate cancer prognosis. They found that there were many causes for sleep disruptions in prostate cancer, including hormones, key genes, diet, inflammatory processes and distress. In cluster #9, research on preventing the suppression of melatonin was discussed. This proves particularly important, as in Porcacchia et al. [82]’s review, the data showed the possible anti-inflammatory and immunomodulatory effects of melatonin in prostate tumours. Research into the possible use of melatonin in treatment and an interdisciplinary approach by using sleep medicine professionals in designing treatment protocols were recommended.

The cited articles in this cluster looked at sleep disruptions, chronodisruption, shift work, and prostate cancer risk [83,84,85,86,87,88,89,90]. One cited article written by Fritschi et al. [83] hypothesised various mechanisms that linked shift work and cancer. These included reduced production of melatonin associated with light, phase shift, sleep disruptions, lifestyle factors and lower levels of vitamin D. Sigurdardottir et al. [84] conducted a case-cohort study looking at urinary melatonin levels, sleep disruption, and prostate cancer risk in elderly men. They found that men who reported sleep disruptions at baseline had lower levels of morning-void urinary 6-sulfatoxymelatonin (aMT6). When compared to males with aMT6 levels above the median in this trial, men with morning aMT6 levels below the median had a fourfold statistically significant increased likelihood of developing advanced disease. To determine whether these findings can be replicated, similar research must be conducted in larger populations with longer follow-up periods. Although there is a clear link between sleep disruptions due to shift work and prostate cancer, further research is needed to understand the processes underlying this link and potential treatments to mitigate the negative impacts of shift work.

### 4.6. Cluster #9: Chronodisruption, Light Exposure, and Cancer

The major citing articles in this cluster were written by Kecklund and Axelsson [91], Itani and Kaneita [92] and Erren et al. [93]. They had a GCS of 530, 9 and 44, and coverages of 9, 7 and 4, respectively. This cluster focused on the relationship between shift work, chronodisruption, sleep, and cancer. In their review, Kecklund and Axelsson [91] discovered that acute sleep loss was the primary impact that shift work had on sleep, particularly in the context of early and night shifts (relative risk range 1.01–1.32). This review identified the need for more research to determine whether insufficient sleep from shift work is a causal pathway for adverse health effects such as cancer. Itani and Kaneita [92] looked at the adverse effects of shift work and found that many studies found an association between shift work and cancer, particularly breast and prostate cancer. In their review, they found that whilst some studies found that there was a link between prostate cancer and shift work, other studies found insufficient evidence to prove a link. In 2007, shift work, which interferes with circadian rhythm, was categorised by the International Agency for Research on Cancer (IARC) as probably carcinogenic to humans. Erren et al. [93] summarised the reasons for this classification and provided 10 theses that highlight the need for additional study to create effective solutions. Some of the theses presented were the need to determine whether there is a causal relationship between chronodisruption and cancer, if so, what component of chronodisruption and shift work increases a person’s susceptibility to cancer, and what preventive measures might be taken. The risk between chronodisruption and prostate cancer was specifically investigated in a few of the cited articles in this cluster [94,95]. Sigurdardottir et al. [94] evaluated epidemiological research on circadian disruption and the risk of prostate cancer. They discovered that while the hypothesis that disruption of the circadian rhythm can raise the risk of cancer is plausible, a more thorough investigation is required before any firm conclusions can be made.

Shift work was found to disrupt the circadian rhythm; therefore, preventative or risk-reducing therapies are required. The role of melatonin and light exposure in circadian disruption was examined in additional cited articles in this cluster [96,97,98,99]. Human melatonin production is suppressed by bright lighting, particularly by shorter light wavelengths, such as those under 525 nm [97]. According to research by Brainard et al. [99], light with a wavelength between 446 and 477 nm was the most potent in melatonin regulation. A study on the usage of eyewear that blocks shorter wavelengths of light was conducted by Kayumov et al. [97]. They looked at the impact of this eyewear on melatonin production and job performance. Nineteen people were given glasses that block all light waves shorter than 530 nm, and blood melatonin levels were assessed. All participants who wore the glasses maintained their melatonin levels; however, unfiltered bright light greatly decreased melatonin levels. The subjects’ performances during their shifts in this study were unaffected. Despite being a small study, it raises the idea of using light-based therapy to help regulate shift workers’ circadian rhythms.

### 4.7. Cluster #19: Testosterone Therapy in Elderly Men

There were two major citing articles in cluster #19. One was written by Basaria and Dobs [100] and had a coverage of nine articles and a GCS of 87. The second article was written by Lund et al. [101], which had a coverage of three articles and a GCS of 71. The focus of this cluster was androgen therapy in elderly men and its possible effects. Although this cluster does not focus on cancer, it introduces the possible effects that hormone therapy can have in men, which can include fatigue. Basaria and Dobs [100] found that testosterone therapy was linked to polycythemia and sleep apnoea. Androgen therapy is contraindicated in prostate cancer. As this cluster does not focus on cancer or the hormone treatments given in cancer, it will not be discussed in detail.

### 4.8. Cluster #6: Hot Flashes

There were two major citing articles in this cluster. The first one was written by Shanafelt et al. [102], which had a coverage of 16 articles and a GCS of 224. The second one was written by Stearns et al. [103], which had a coverage of 15 articles and a GCS of 340. The majority of cited papers in this cluster concentrate on the causes and treatments of hot flashes either in relation to cancer or menopause. Shanafelt et al. [102] and Stearns et al. [103] discuss hot flashes in regard to menopause and recommend hormone therapy or specific SSRIs or SNRIs, such as venlafaxine, as the best therapies. Stearns et al. [103] briefly discuss prostate cancer in their review. They discovered that although it was generally known at the time that men undergoing androgen-deprivation therapy experienced hot flashes, the severity, and other symptoms such as changes in sleep and mood, had not been thoroughly examined. A number of cited articles in this cluster looked at prostate cancer and hot flashes [104,105,106]. Holzbeierlein et al. [104] conducted a review on the side effects of ADT. They found that up to 80 percent of patients on LHRH agonists will experience hot flashes. This review also looked at possible treatments which included antidepressants, hormone treatments, and alternative therapies. Quella et al. [105] and Loprinzi et al. [106] found that venlafaxine was an efficient way to control hot flashes in men undergoing androgen-ablation therapy for prostate cancer. Hot flashes had a significant impact on the quality of life of men undergoing ADT. Holzbeierlein et al. [104] recognised the need for counselling patients about potential side effects as well as the need for more research, at the time, into the extent of ADT side effects and potential treatments.

### 4.9. Cluster #33: Management of Testosterone Deficiency

The main citing articles in this cluster were written by Burté [107] and Bhasin and Ozimek [108]. They had a GCS of 4 and 3, and a coverage of 5 and 3, respectively. These papers focused on diagnosing and treating testosterone deficiency. Cited articles in this cluster looked at the effects of long-term testosterone therapy. As this cluster does not discuss prostate cancer or sleep, it will not be discussed in detail.

### 4.10. Cluster #24: Androgen Deprivation Therapy

The main citing articles in this cluster were written by Østergren et al. [109], Rot et al. [110], and Dimopoulou et al. [111]. They had a GCS of 20, 4, 38 and a coverage of 7, 4, and 2, respectively. This cluster focuses on the negative consequences of ADT and potential solutions. The advantages of exercise to combat the negative effects of ADT were evaluated by Østergren et al. [109]. The impact of ADT on bone loss, cardiovascular disease, metabolic consequences, and fatigue were the main topics of discussion. Exercise was found to reduce fatigue rates, but there was insufficient data to support its use in treating ADT’s other side effects. In a study comparing urologists in Canada and lower-GDP nations, Rot et al. [110] sought to determine what urologists believed to be critical in ADT patient education. The sole side effect that was more commonly disclosed to patients in the other nations was infertility problems, whereas Canadian urologists were more likely to mention fatigue, hot flashes, and sleep disturbances to their patients. Several cited articles examined lifestyle interventions to manage ADT adverse effects [112,113,114]. Although they had to be maintained, lifestyle interventions had positive impacts on fatigue while they were being used but had little impact on the other side effects of ADT.

### 4.11. Cluster #0: Androgen Deficiency and Cluster #2: Diagnosis and Treatment of Hypogonadism

Research on androgen deficiency was addressed in cluster 0, and treatments for hypogonadism were discussed in cluster #2. Gooren [115] and Tung and Cunningham [116] wrote the major citing articles in cluster #0. They had respective coverages of 23 and 17 and GCSs of 19 and 5. The primary citing articles in cluster #2 were written by Buvat et al. [117] and Bhasin and Basaria [118]. They both had a coverage of 11, and their respective GCSs were 195 and 69. These clusters will not be discussed, as they do not pertain to prostate cancer or sleep.

## 5. Conclusions

Some limitations need to be considered to correctly interpret the results from the current scientometric review. First, the results highly depend on the initial documents identified on Scopus. It is possible that some relevant documents that are not indexed in Scopus might have not been included in the sample of citing documents. Furthermore, a method that relies on the quantitative analysis of the patterns of co-citations between documents inevitably provides clearer light on past publications and research trends rather than on more recent ones [14]. Some recent trends of research with some initial studies did not emerge as frequently to be identified as major thematic domains in the literature. Nonetheless, the current scientometric review article has highlighted some important themes with regard to prostate cancer and its effect on sleep patterns, with some reports showing sleep disturbance and sleep disorders.

Whilst the mechanisms underlying sleep disorders in patients with cancer are yet to be fully understood, there is a notable disparity between the frequent occurrence of disruptive sleep in men with prostate cancer and the lack of randomized scientific trial data assessing the impact of treatment and therapeutic interventions. This is in contrast to epidemiological data postulating that there may be a link between poor sleep and the development of prostate cancer, although how sleep and the development of cancer are related yet to be understood.

It is well recognised that low levels of testosterone are associated with self-reported insomnia and fatigue, and this provides a plausible rationale for the effect of ADT and its suppression of testosterone in causing a disruption of sleep quality and fatigue, over and above that of the fear and anxiety associated with a cancer diagnosis. However, testosterone suppression does not appear to be the sole driver for the fatigue and insomnia observed in prostate cancer patients, as other local treatments such as radiotherapy (external beam or brachytherapy) and surgery are also contributory, suggesting that an unbalance in the level of inflammatory cytokines such as interleukins and tumour necrosis factor-alpha (TNF-alpha) are involved in dysregulated sleep patterns [119,120]. Furthermore, symptoms such as sweats/hot flashes and frequent nocturia may also be contributing to overall reduced sleep quality.

However, a key limitation of the studies on sleep and prostate cancer is that the distinction between cancer-related fatigue (CRF) and sleep disorder such as insomnia are infrequently made and are not precise in terms of sleep diagnosis. Although CRF and insomnia are commonly interrelated, with one impacting the other, it is important nevertheless to make the distinction. CRF implies overwhelming tiredness, exhaustion, and weakness that does not go away with sleep and rest (i.e., assumes that you have a normal sleep pattern), whereas a sleep-related disorder such as insomnia is more specific and can be determined from validated questionnaires, such as the Insomnia Severity Index (ISI) or Pittsburgh sleep score, or more objectively with actigraphy. This review has highlighted a paucity of data with regard to disruption of sleep patterns in prostate cancer patients, relying mainly on patient-reported outcomes on fatigue and its impact on quality of life, rather than using validated sleep questionnaires, with only a handful of studies utilising some form of objective assessment of sleep quality with actigraphy or polysomnography to assess sleep disturbances. Even fewer studies have some form of baseline assessment prior to starting treatment to fully ascertain the effect of hormonal treatment on sleep quality or quantity. At present, the majority of phase-three clinical trials in prostate cancer, whilst collecting subjective data on cancer-related fatigue through the health-related quality of life outcomes, have very limited information on sleep disturbance derived from the FACT-P questionnaire, having just one question that addresses sleep quality in the functional well-being section. The collective use of actigraphy and subjective sleep questionnaires is both feasible and relatively inexpensive in a clinical setting to assess sleep profiles that can serve as a platform for conducting larger clinical trials. This has been corroborated in our own institutional feasibility study of 74 patients with prostate cancer [121].

The identification and management of a sleep disorder such as insomnia are important since they may be amenable to the use of behavioural and/or pharmacological intervention as noted in this review, thus having the potential not only to improve the quality of life for patients with prostate cancer but also treatment compliance. Moreover, mapping out the multi-factorial nature of sleep disorders in patients with prostate cancer is also essential in relation to well-known vasomotor symptoms such as hot flashes/sweats and nocturia for targeted and individualised management to improve sleep quality.

This review did not show clusters that investigated whether a different hormonal agent may have a differential effect on sleep disturbance, specifically with regard to whether anti-androgens (which maintain testosterone levels), transdermal oestrogens, and the newer androgen receptor-targeted agents such as Enzalutamide, Abiraterone, or Daralutamide, report fatigue as a recognised side effect. This is clearly an unmet need and is worthy of further investigation. In brief:

- Sleep disturbances and vasomotor symptoms associated with androgen deprivation therapy affect the quality of life for men with prostate cancer.
- Much of the data rely on subjective rather than objective assessment of sleep quality and hot flashes.
- The subjective data do not seem to be able to distinguish between cancer-related fatigue or a sleeping disorder, such as insomnia.
- Actigraphy has been used in clinical settings as a non-invasive method of characterising sleep, but studies thus far have not explored whether sleep quality varies according to the type of androgen-deprivation therapy.
- The limited use of actigraphy is hindered by the lack of use of baseline assessments. This is particularly important given that sleep disturbances are generally considered multifactorial.

The smaller-scale studies outlined here show that the use of sleep devices such as actigraphy together with questionnaire assessments is a feasible way of assessment of sleep disturbances in a clinical setting. With an increasing number of systemic treatments for prostate cancer being introduced into the clinical setting that are likely to have similar clinical efficacy, with a requirement to use them until disease progression, it is important to develop a sleep assessment for this population using a combination of subjective and objective tools. Establishing such a platform would be key in developing clinical trials for prostate cancer patients in the future.

## Figures and Tables

**Table 1:** Top ten documents with citation burstsness.

References	Citation Burstness	Publication Year	Burst Begin	Burst End	Duration	Centrality	Sigma
Scher et al. [39]	9.5844	2012	2013	2019	6	0.04	1.45
Bhasin et al. [40]	8.4489	2010	2012	2015	3	0.01	1.07
Beer et al. [41]	7.7534	2014	2015	2023	8	0.04	1.32
Basaria et al. [42]	7.7256	2010	2011	2016	5	0.20	4.07
Sih et al. [43]	7.6022	1997	1999	2005	6	0.02	1.21
Harman et al. [44]	6.7064	2001	2002	2008	6	0.05	1.38
Ryan et al. [45]	6.4786	2013	2014	2019	5	0.00	1.02
Amory et al. [46]	6.2562	2004	2005	2010	5	0.04	1.31
Snyder et al. [47]	5.9516	1999	2003	2006	3	0.00	1.01
Bhasin et al. [48]	5.8709	2006	2007	2010	3	0.02	1.10

**Table 2:** Summary metrics for the 12 major clusters identified in the document co-citation analysis network.

Cluster ID	Size	Silhouette	Mean Year	LLR Label	Suggested Label
0	100	0.969	2002	clinical practice guidelines	androgen deficiency
1	58	0.931	2013	metastatic castration-resistant prostate cancer	metastatic castration-resistant prostate cancer
2	50	0.990	2008	testosterone deficiency	diagnosis and treatment of hypogonadism
3	35	0.967	2015	prostate cancer risk	night work and prostate cancer risk
5	26	0.985	2000	cross-sectional comparison	insomnia and fatigue in cancer patients
6	24	0.996	2000	hot flashes	hot flashes
9	23	0.981	2009	health consequence	chronodisruption, light exposure, and cancer
10	19	0.977	2010	oncological treatment	assessment of circadian rhythms in oncology treatments
19	11	0.990	1994	elderly men	testosterone therapy in elderly men
24	9	0.941	2012	androgen deprivation therapy	androgen deprivation therapy
25	7	0.998	2015	preference	sleep management
33	6	1.000	2017	practical recommendation	management of testosterone deficiency
